# Trends and Perspectives in Immunosensors for Determination of Currently-Used Pesticides: The Case of Glyphosate, Organophosphates, and Neonicotinoids

**DOI:** 10.3390/bios9010020

**Published:** 2019-02-04

**Authors:** Eduardo C. Reynoso, Eduardo Torres, Francesca Bettazzi, Ilaria Palchetti

**Affiliations:** 1 Posgrado en Ciencias Ambientales, Instituto de Ciencias, Benemérita Universidad Autónoma de Puebla, Puebla 72570, Mexico; eduardoc.reynoso@gmail.com (E.C.R.); eduardo.torres@correo.buap.mx (E.T.); 2 Dipartimento di Chimica, Università degli Studi di Firenze, Via della Lastruccia 3, 50019 Sesto Fiorentino (Fi), Italy; francesca.bettazzi@unifi.it

**Keywords:** immunosensor, pesticides, glyphosate, organophosphates, carbamates, neonicotinoids

## Abstract

Pesticides, due to their intensive use and their peculiar chemical features, can persist in the environment and enter the trophic chain, thus representing an environmental risk for the ecosystems and human health. Although there are several robust and reliable standard analytical techniques for their monitoring, the high frequency of contamination caused by pesticides requires methods for massive monitoring campaigns that are capable of rapidly detecting these compounds in many samples of different origin. Immunosensors represent a potential tool for simple, rapid, and sensitive monitoring of pesticides. Antibodies coupled to electrochemical or optical transducers have resulted in effective detection devices. In this review, the new trends in immunosensor development and the application of immunosensors for the detection of pesticides of environmental concern—such as glyphosate, organophosphates, and neonicotinoids—are described.

## 1. Introduction

Every year, millions of tons of pesticides are produced for agricultural purposes [1]. Due to this high demand, there is a toxicological risk that strongly impacts on human health [2], and on the ecosystems [3,4]. According to the World Health Organization (WHO) and the Food and Agriculture Organization (FAO) a pesticide is *“*any substance, or mixture of substances, or micro-organisms including viruses, intended for repelling, destroying or controlling any pest, including vectors of human or animal disease, nuisance pests, unwanted species of plants or animals causing harm during or otherwise interfering with the production, processing, storage, transport, or marketing of food, agricultural commodities, wood and wood products or animal feeding stuffs, or which may be administered to animals for the control of insects, arachnids or other pests in or on their bodies” [5].

Pesticides can be classified according to their chemical structure (i.e., organochlorines, organophosphates, carbamates, pyrethroids, etc.) [2,6], the associated toxicological risk (extremely, highly, moderately, slightly hazardous) [7], the persistence (permanent, persistent, moderately persistent, and not persistent) [8] or their toxicity according to their chemical structure, mechanism of action, and fate [9]. The most common classification is according to the use or type of target pest (insecticides, herbicides, fungicides) [6].

Pesticides are applied mainly by spraying directly to the plants or to the soil. Therefore, they can also reach other environmental compartments, such as the atmosphere, the surface, and underground waters where they can be absorbed by aquatic and terrestrial biota [10,11] among birds, fish, and mammals [12,13,14,15]. It is very complicated, currently, to reduce the use of pesticides [1] and the implementation of mitigation strategies [4,11,16]. The rapid determination and continuous monitoring of both pesticides and their metabolites is an extremely important point to guarantee the respect of the legal limits [8]. To ensure the health of the population that is in contact with contaminated food and of the farmers themselves [17], the World Health Organization (WHO) has established the Codex Alimentarius [18] which includes the Maximum Residual Levels (MRLs) that must comply with all pesticides for different foods. The MRLs are “the highest level of a pesticide residue that is legally tolerated in or on food or feed when pesticides are applied correctly in accordance with Good Agricultural Practice and applies to the 303 pesticides listed by FAO” [19].

There are several analytical methods to detect pesticides in environmental and food samples. Most of them are based on gas and liquid chromatography coupled to mass spectrometry (MS and tandem MS) [20,21]. Using these techniques low detection limits can be achieved obtaining results with high selectivity and accuracy. However, this kind of analysis should be performed necessarily by well-trained personnel due to the laborious sample treatments [22,23] and the complex analytical procedures. Therefore, despite the associated high costs and low throughput, these kinds of techniques are undoubtedly suitable as a confirmatory method. However, for a massive monitoring program, the development of screening analytical methods to be performed directly in the field is mandatory. Screening methods allow the use of confirmatory method only on positive findings, thus decreasing the number of samples to be analyzed reducing the cost. Indeed, screening methods must be easy to perform, high throughput, cost effective, with adequate sensitivity and low rates of false negatives. Screening techniques for detecting trace amount of chemicals for both laboratory and field analysis are usually provided by biochemically based assays. Among the high number of biochemical assay techniques, enzyme linked immunosorbent assays (ELISAs), combined with a colorimetric measurement, are the most widely used. The use of biosensor technology is an alternative approach which possesses technical simplicity, low cost, and suitability for field analysis [24]. Biosensors can be taken to the sampling sites, having results in real time [8,25,26,27]. Several reviews have shown different types of biosensors for the detection of pesticides in recent years [8,24,28,29,30,31,32,33,34].

Among the different types of biosensors for pesticide determination, immunosensors [29] have gained in popularity in the last decade [35,36,37,38] as rapid screening techniques because of specificity, sensitivity, low cost, and suitability for high throughput screening.

In this paper, recent trends in immunosensor and immunoassay development are addressed, with a special focus on the new trends in antibodies production and selection towards environmental contaminants. Different optical and electrochemical immunoassay schemes currently reported for a selected number of pesticides (glyphosate, carbamate, organophosphate, and neonicotinoids) are reviewed, focusing on a literature survey of the last five years (2013–2018).

## 2. Immunosensor Development: Practical Tips and New Trends

Antibody-based biosensors (immunosensors) are important examples of affinity biosensors [39]. As already stated, immunosensors depend on a reaction between an antigen (the analyte) and an antibody (the bioreceptor). By definition, an antigen is a molecule that elicits an immune response (immunogenicity) and is capable of combining with the specific antibody. Molecules that bind to antibodies but that do not possess immunogenicity are known as ‘haptens’. Pesticides are typical haptens that can induce the immune system to produce antibodies only when attached to a larger carrier such as a protein. Common carrier proteins are bovine serum albumin and keyhole limpet hemocyanin, among others.

Antibodies are the most used affinity proteins for all life science applications and are the key reagent in the development of an immunosensor. The main characteristics of an antibody are the high specificity and affinity toward the target. The IgG molecule, which is the most used antibody type, is a ≈150 kDa protein composed of four polypeptide chains, two identical shorter light chains (VL) and two identical larger heavy chains (VH). A light chain is coupled to a heavy chain via a disulfide bond and the two disulfide-bridged dimers correspondingly form the typical Y-shaped antibody structure. Antibodies treated with papain protease generate two identical antigen-binding fragments (Fab, fragment antigen binding) and one without antigen binding activity (Fc, fragment crystallizable). The Fab is formed by two domains from each light and heavy chain of the antibody: the variable domain, also known as the FV region, and one constant domain. The variable FV domain is the most important region for binding to antigens. Antibodies can be classified as polyclonal (PAb), monoclonal (MAb), and recombinant (RAb) antibodies. PAbs are produced by different cellular clones as a result of the animal immunization and are extracted from the living animal serum; PAbs recognize different epitopes on the same antigen and are polyspecific. MAbs are produced by hybridoma technology, using cell fusion from splenic and tumor cells; splenic cells originate from immunized animals and are fused to immortalized cancer cells to provide endless production of the selected MAbs. MAbs, combining to a single epitope on the antigen, are monospecific and more homogeneous than PAbs. However, monoclonal antibody production is laborious. Thus, the technology to produce optimized biorecognition elements has led to the development of RAbs. RAbs are monoclonal antibodies generated in vitro using synthetic genes; they are produced in bacterial cell using phage display technology. Antibody genes are obtained from source cells and undergo amplification and cloning into an appropriate phage vector; the vector is introduced into a host (bacteria, yeast, or mammalian cell lines) to achieve the expression of adequate amounts of functional antibodies. Display libraries are commonly expressed in phage or yeast; libraries could be properly analyzed to select for desirable characteristics in the antibody sequence. The obtained antibodies are encoded, selected, and expressed in multiple structural formats, which can each have considerably different biophysical attributes. The simplest and commonly used structural format is the single chain fragment (scFv) in which the variable-domains of antibodies are linked by a flexible peptide linker.

Nowadays, many antibodies are commercially available for several individual molecules among the different classes of pesticides including organophosphorus-, carbamate-, organochlorine-, pyrethroid-, triazine-pesticides, and others. Binding specificity is a key feature of an immunoassay. However, depending on the conjugate used for immunization and the class of chemicals under study, cross-reactivities of the antibody with haptens similar to the analyte are commonly observed. For single target immunoassay, cross-reactivity could be a problem and could generate false positive values. This problem can be controlled by specific sample treatment procedures. However, for screening purposes and for high throughput sample analysis, could be useful to perform a multiplexed analysis (more than one target analyte simultaneously). A novel trend in the production of antibodies for pesticide determination is to raise a single antibody capable to determine several analytes in one test, which is called broad-specificity antibody. A strategy for broad-specificity antibody production is to synthesize generic hapten based on similar structure of a group of molecules, and then to prepare an antibody with broad cross-reactivity. Antibodies with broad detection spectrum are an attractive approach for multi-residue monitoring. As an example, Jiao et al. reported the development of broad-specific monoclonal antibodies against parathion, methyl-parathion, and fenitrothion (organophosphorus pesticides) [40].

Recently, nanobodies (Nbs), single-domain antibody (sdAb) fragments formed by the variable domain of antibodies found in camelids (e.g., dromedaries, llamas, alpacas) or in non-mammals, such as sharks and ratfish, have been proposed for biosensing [41]. These single-domain antibodies have molecular weights ranging between 12 to 14 kDa and constitute the smallest domains of natural antibodies with antigen-binding capacity. Nbs can be produced in bacteria and yeast with less cost than conventional monoclonal antibodies and are encoded by a single gene segment. Moreover, recent studies demonstrated that nanobodies could be particularly suited for the development of immunosensor because can be easily modified by site-specific functional groups, obtaining covalent and oriented binding with a negligible loss of specificity and affinity. Recently, different Nbs have been discovered and applied for environmental contaminant determination [42]. Sun et al. reported the selection of a Nb against ochratoxin A in cereal and the development of a Nb-based ELISA. The limit of detection (LOD) reported for the Nb-based ELISA was of 1.4 μg/kg in barley, 0.56 μg/kg in oats, and 0.84 μg/kg in rice [42]. Tang et al. reported the development of a fluorescent assay using Nbs selected against aflatoxin and zearalenone in maize and in maize-containing products [43]. A Nb-alkaline phosphatase was recently developed for the determination of 3-phenoxybenzoic acid (a human urinary metabolite of many pyrethroid insecticides) in urine [44].

Moreover, the increasing experience in the field of combinatorial libraries and protein engineering has inspired researchers to develop new non-immunoglobulin affinity proteins. Consequently, a large number of engineered protein scaffolds [45], such as affibodies [46], are nowadays commercially available. However, to the best of our knowledge, these new molecules are mainly produced for clinical application. Bacteriophages are another promising alternative to traditional antibody-based molecules [47].

**Figure 1:**
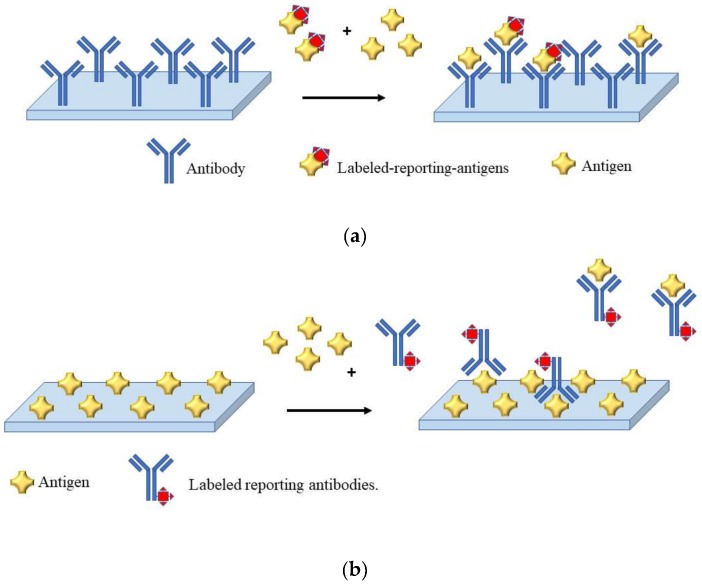
Schematic representation of a competitive immunoassay. (**a**) The antibody is immobilized; (**b**) the antigen is immobilized.

In the development of an immunosensor, the sensing strategy is carried out using competitive and non-competitive assay format; usually, the choice is made on the basis of the molecular size and reactivity of the analyte. Thus, for analytes holding two or more epitopes the non-competitive approach is used, whereas for smaller or less reactive analytes a competitive approach is generally preferred. In the case of pesticide analysis, the assay is mostly performed using a competitive assay scheme, since pesticides are relatively small molecules. In a competitive scheme, the magnitude of the response is inversely proportional to the concentration of the analyte (sample antigen). Figure 1 is reports a scheme of a competitive assay. In the format illustrated in Figure 1a, the molecules of the antigen in the sample compete with the labeled-reporting-antigens added in the sample for binding with the immobilized antibodies. In the competitive format illustrated in Figure 1b, the antigens in the sample compete with the immobilized antigens for binding with the labeled reporting antibodies. For both formats, several transducing techniques are possible. Herein, the discussion will be focused on electrochemical (i.e., potentiometric, amperometric, voltammetric, and impedimetric) and optical (surface plasmon based (SPR), colorimetric, fluorescent) immunosensors for pesticide determination since are the most used. However, we ask the reader to refer to detailed book and review paper for the theory and the setup of other possible techniques [48]. Immunosensors can be label free or label-based. Enzymes, nanoparticles, or fluorescent dyes are the most common labels [49,50,51].

The immobilization procedure is crucial in the development of an immunosensor. The most common materials of optical and electrochemical transducer surface are gold, glass, metal oxides, carbon (i.e., glassy carbon, graphite, graphene, etc.). Both antibody and antigen can be immobilized on the transducer surface. Different immobilization procedures can be used. Physical adsorption is mainly based on the non-covalent (mainly electrostatic) interaction between the antibody and the transducer surface as well as the entrapment into a three-dimensional network of gel or polymers (including conducting polymers). Covalent chemical binding via functional moieties naturally present on the surface or ad hoc generated is another possibility. The use of ordered layers on the transducer surface is a common practice in order to have an oriented immobilization and achieve a better presentation of the antibody to the target analyte. This can be obtained by deposition of self-assembled layers (SAM) of thiols on gold or by modification of the transducer surface by carboxyl-confined-, silanized-, or aldehyde-derivatives. The immobilization procedure of SAMs, through thiol-gold chemistry, usually involves two separate steps. In the first one, the SAM is created in order to obtain a convenient reaction with the antibody. To this end, the functionalities of a spacer containing a thiol at one end and a functional group (mainly carboxylic acid and amine) at the other end were exploited. The second step involves the reaction of the antibody with the functional group of the spacer, through covalent bonds (such as amide bonds and Schiff’s base formation), directly or by the aid of bridging molecules such as glutardialdehyde [36]. Carbodiimide and hydroxysuccinimide derivatives have also found several applications. The obtained layer is well oriented on the transducer surface thus allowing a better antibody/analyte interaction. Moreover, the optimal average spacing between the receptors can be controlled thanks to the use, for SAMs preparation, of a mixed solution containing both the recognition element and a spacer (usually a short chain alkane-thiol) obtaining the best performance of a well-behaving electrochemical surface. An innovative method to prepare SAM is by using aryl diazonium salts. Aryl diazonium salts have become particularly attractive for sensing due to recent reports that indicate that this chemistry can be applied to, not only all sorts of carbon surfaces (glassy carbon, graphite, screen printed electrodes carbon nanotubes, and diamond), but also metals, metal oxide, silicon. Thus, it appears as a modification chemistry that can be used for most, if not all, type of transducer surface [52,53].

Moreover, instead of being immobilized on the sensor surface, antibodies can be immobilized on a separate support (magnetic micro- and nanobeads). In these cases, the transducer is used only for the measurement. This approach is generally performed with electrochemical transducers [54,55,56]. Immunomagnetic assays are believed to be particularly useful for the improvement of the analytical performance of the electrochemical immunoassays, since the immunoreaction is performed on a different surface, with respect to electrochemical detection.

Regarding the transducers, the present report describes only optical and electrochemical transducers. Electrochemical immunosensors are based on monitoring electro-active species that are involved in the reaction of the biological element, which imparts biofunctionality to these structures, with the analyte. The different detection methods can be obtained using three main quantities: current (amperometric and voltammetric biosensors), potential and impedance [48]. Several examples of papers in which this kind of detection strategies are exploited, will be discussed in further paragraphs. In particular, the amperometric detection rely on the constant application of certain potential to the electrochemical cell and measuring the current [57,58]. Whereas, the voltammetric techniques rely on the application of a varying potential, in a specific range, on the monitoring a current response under controlled potential conditions [59]. Thus, the signal due the presence in solution of the analyte with a specific redox behavior can be detected. In both amperometric and voltammetric detection schemes, the current can be correlated to the concentration of electroactive species in solution. In potentiometry, the information about the composition of a sample is obtained through the passive measurement of the potential between two electrodes, namely the indicator and reference electrode. The exploitation of electrochemical impedance spectroscopy (EIS) detection is particularly useful for label-free immunosensors. EIS technique is based on the observation of the system upon the perturbation of the cell with an alternating signal of small magnitude, overlaid on a constant DC bias potential, commonly as a function of the frequency [48]. The frequency is typically varied over a wide range to obtain the impedance spectrum. In the most popular EIS method, classified as Faradaic, measurements are performed in the presence of a redox couple in solution, which can amplify the changes occurred during biorecognition event. The analytical data can be analyzed using theoretical model circuit where the electron transfer resistance (R_et_) is employed as a main indicator. This is because the R_et_ values indicate the variation of the impedance at the electrode surface upon modification with a capture molecule. Moreover, with this approach it is possible to measure the modification of R_et_ caused by the selective capturing of a given analyte. The electrode surface can be also modified using nanomaterials to develop biosensing platforms with enhanced electrochemical features. This approach has been successfully exploited in the development of biosensing platforms for pesticide detection [60]. A novel interesting electrochemical approach is the photoelectrochemical (PEC) technique. The PEC transducer could be roughly described as a photoactive material in contact with a conductive substrate. The antibody, immobilized onto the transducer surface, acts as the biorecognition molecule and recognizes the target antigen. PEC immunosensors convert the affinity reaction into a detectable electrical signal, via excitation and charge transfer of a photoactive material upon absorption of light [61,62,63]. PEC detection offers some peculiar advantages of the electrochemical detection, i.e., low cost, simple and easy to miniaturize instrumentation, etc. Furthermore, by using light for excitation and a separate form of detection, PEC possesses potentially high sensitivity because of the reduced background associated with it.

Other intriguing approaches that can be developed in the optimization of real time and label-free affinity-based biosensing rely undoubtfully in the use of optical detection by surface plasmon resonance (SPR) [40,64]. SPR based biosensors belong to refractometric devices and have demonstrated to be user friendly, versatile, thanks also to the recent development of low-cost devices. SPR-based biosensors are very powerful tools for the study of biomolecular interactions, in immunoassays. The detection scheme is based on the evaluation of the surface optical properties. In particular, the interaction of the (bio)recognition element with the target analyte causes a variation in the refractive index at the interface that results in a change of the resonance angle. This variation can be correlated to a real time and label-free signal.

Plasmonic nanomaterials (PNs) display interesting optical properties leading to an increasing interest in the field of optical transducer. LSPR spectrophotometric measurements can be achieved by means of several optical substrates. The wavelength shifts of the absorption band or the color change of the nanoparticle solution is the results of the contribution of the oscillations of localized plasmons at the single PNs interface. Thus, these phenomena can be correlated with the dispersion of nanoparticles (NPs) in solution. Bioluminescence, chemiluminescence, fluorimetry, and colorimetry are other label-based techniques widely used in the development of optical biosensors.

## 3. Immunosensors for Pesticide Determination

Among the different pesticides, glyphosate is the most used herbicide worldwide, whereas the organophosphate chlorpyrifos is one of the most used insecticides. Thus, in the following sections some examples of immunosensors against these types of pesticides are reviewed. A focus on immunosensors for determination of neonicotinoids (a class of new generation pesticides) is also reported.

### 3.1. Glyphosate

Glyphosate (N-phosphonomethyl-glycine) is an organophosphorus herbicide of broad-spectrum and systemic action. It was introduced in the early 1970s by Monsanto under the name of Roundup [65]. Glyphosate is involved in biochemical alterations of processes of microorganisms and plants, inhibiting the enzyme 5-enolpyruvylshikimate-3-phosphate synthase (EPSPS) responsible for the biosynthesis of aromatic amino acids; the decrease of these amino acids in the organism reduces the synthesis of proteins causing the cessation of growth and consequently the death [66]. It is sprayed on millions of hectares worldwide, being the most used herbicide on the globe. This mainly is due to the increase, in recent years, in the sale of genetically modified organisms (GMOs) resistant to the activity of this herbicide and the ease of combining with other pesticides [67,68].

A debate whether glyphosate is carcinogenic or not [69,70,71] is still present, since, the International Agency for Research on Cancer (IARC) established in 2015 [72] that glyphosate is a “probable carcinogen to humans” classifying it in group 2A. However, recently the European Food Safety Authority (EFSA) and the European Chemicals Agency (ECHA) concluded that there is not enough evidence that glyphosate represents a carcinogenic hazard [73,74]. Nevertheless, it is very important to determine its presence in environmental, food, or biological samples. There are several worldwide standards on the maximum concentrations allowed. FAO establishes the MRL for human food of 0.05 mg/kg in the case of milk and egg; 5 mg/kg for maize, lentil, peas; and 20 mg/kg for soya bean and wheat bran, for some examples [75]. In the case of drinking water, the European Union establishes a maximum level of 0.1 μg/mL for this herbicide [76]. In the United State of America (USA), the United State Environmental Protection Agency (USEPA) establishes the maximum level of 700 μg/mL [77]. The disparity among the concentrations is extremely high, even in countries close to US such as Mexico (10 μg/mL) [78] and Canada (280 μg/mL) [79]. This is due to the legislation on the use of GMOs in the USA and the amount of hectares of soil where this herbicide is applied [67].

The USEPA official method to detect glyphosate in drinking water [80] involves liquid chromatography coupled to fluorescence detectors; this procedure applies to USA regulations so that its sensitivity is not low enough to detect the minimum concentration in the EU. Immunosensors are claimed to be interesting tools to detect this substance at low concentration and without the need for laborious sample treatment processes. In the following sections, detection in different foods, soil, and water will be addressed by electrochemical and optical immunosensors.

#### 3.1.1. Optical Immunosensor

There are several works about the detection of glyphosate using the immunoassays, the vast majority is based on the enzyme-linked immunosorbent assay (ELISA) [81,82,83,84,85,86]. Gonzalez et al. [87] reported an optical immunosensor that has advantages over conventional immunoassays—such as rapid response, full automation, and sensitivity—and it can be used in field analysis. The LOD was 21 ng/L and the linear range was 50–1000 ng/L, for the analysis of water and soil. The immunosensor is capable of discriminating AMPA and other herbicides such as gluphosinate and glyphosine. Control assays were carried out to check for natural interferences from the soil matrix; results showed that it is necessary to dilute the samples to avoid noise from humic acids, in addition to previous treatment such as alkaline extraction. A colorimetric immunoassay, using gold nanoparticles as labels, was developed by [88], achieving detection of glyphosate in the range of 0.01–100 mg/L. The same assay scheme was proposed in another study by using fluorescence magnetic nanoparticles, consisting of Co–B(core)/SiO_2_ (shell)/dye NPs [89]; LODs in the range of mg/L were reported.

#### 3.1.2. Electrochemical Immunosensor

**Figure 2:**
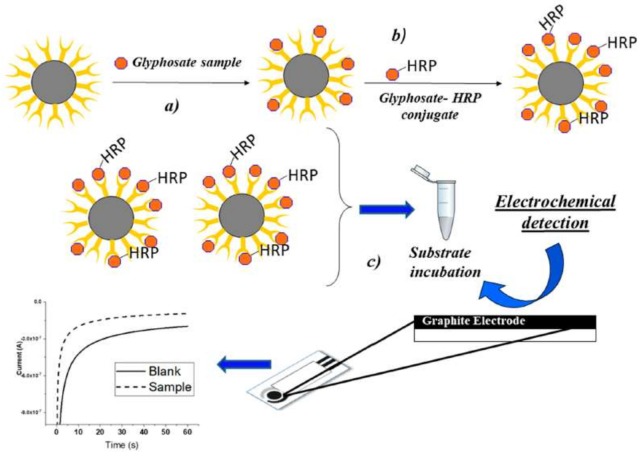
Electrochemical immunosensor for the detection of glyphosate (reprinted with permission from [57]).

Several papers report the electrochemical detection of glyphosate [90,91,92], but only one describe glyphosate detection by electrochemical immunoassay, based on the use of antibody-modified magnetic particles coupled to a disposable screen-printed electrochemical cell [57]. A chronoamperometric measurement of the electroactive product of the oxidation of TMB by the HRP in the presence of H_2_O_2_ (Figure 2) is achieved. This technique shows a better sensitivity with respect to the optical detectors, with LOD of 5 ng/L and LOQ of 30 ng/L for beer-spiked samples.

### 3.2. Carbamates and Organophosphates

Carbamates and organophosphates are a class of insecticides that inhibit the activity of acetylcholinesterase (AChE), an essential enzyme for the functioning of the central nervous system [31,93]. These pesticides are claimed to be less toxic than organochlorines and with a not so prolonged persistence in the environment; in most cases half-lives are less than six months [94]. Despite the fact that the use of these pesticides, in recent years, has decreased by around 50% for some developed countries such as UK and Germany and 70% and in the US, in 2012 alone, almost 32,000 tons of organophosphate insecticides were produced in US [95,96]. This generates great concern in terms of human health because many human consumption crops and livestock animals are potentially contaminated with these pesticides [97,98]. Exposure to contaminated food represents a potential risk in humans [97,99] and mammals [100].

Carbamates are esters of N-methyl carbamic acid and some of the active ingredients in commercial formulations are aldicarb, carbaryl, methyl carbamate, terbucarb, and propoxur. An acceptable daily intake in food below 0.5 mg/kg [101] is generally accepted. Carbamates are classified as non-carcinogenic and are in group 3 of the IARC [102]. In the case of organophosphates, IARC classifies some of them in group 2A (malathion, diazinon) and 2B “possibly carcinogenic to humans” (parathion, dichlorvos, tetrachlorvinphos) [102]. Whereas some other are not classified by IARC including: chlorpyrifos, phosmet, fenitrothion, azamethiphos, disulfoton, and fonofos. The MRL of some organophosphates is greater than for some carbamates (5 mg/kg) in foods as seeds, but in general they remain below 1 mg/kg of dry weight [101]. Because some of them can persist in food for a long time, their toxicity in humans and mammals is high and it is very important to determine their presence at low concentrations [7].

The vast majority of OPs are detected by enzyme-based biosensors, which consist of the enzymatic inhibition of AChE [103,104,105,106]; this inhibition produces a signal based on a decrease in enzymatic activity. These biosensors are very sensitive, but have little specificity [31] and can be affected from many other pollutants, including heavy metals. The use of immunosensors for the determination of these two class of pesticides has grown a lot, due to the great specificity and the possibility to detect individual analytes, besides their high sensitivity, robustness, and reliability [24].

#### 3.2.1. Optical Immunosensor

Jiao et al. [40] developed broad-specific monoclonal antibodies (mAbs) against parathion, methyl-parathion, and fenitrothion, using heterologous indirect competitive enzyme-linked immunosorbent assay (icELISA). A non-competitive surface plasmon resonance (SPR) immunosensor was used for the characterization of the binding properties of the mAbs toward the pesticides, with IC_50_ ranging from 2.93 to 19.71 ng/mL in water samples. To determine the matrix effect, the authors applied icELISA with spiked recovery tests to water samples, without any representative matrix effect.

**Figure 3:**
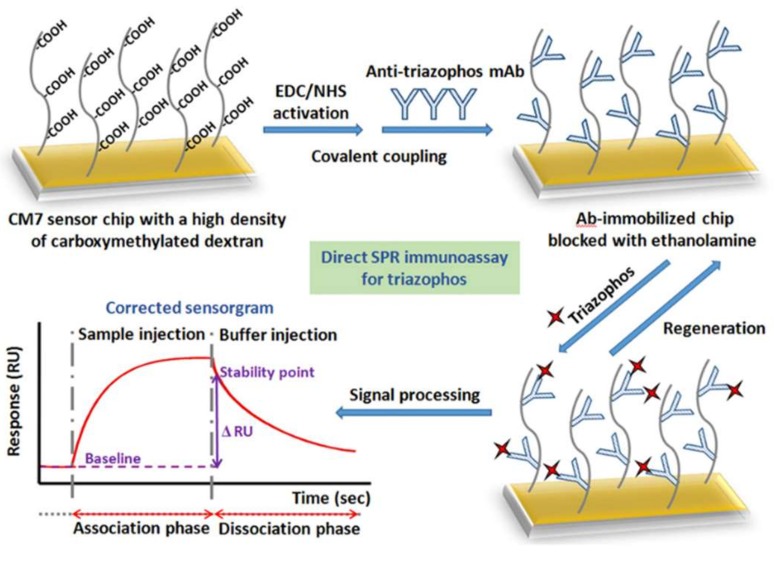
Preparation of Ab-immobilized sensor chip for direct detection of triazophos with real-SPR (reprinted with permission from [64]).

Similarly, a non-competitive SPR immunosensor was developed by Guo et al. [64] (Figure 3) for detection of thiazophos in water and agricultural products, with a LOD of 0.096 ng/mL and a linear range of 0.98–8.29 ng/mL. To evaluate the matrix effect and the non-specific binding to the sensor, the samples from different matrix were spiked with triazophos and analyzed with blank sample control (without fortification of triazophos) with a QuEChERS method. The matrix effect was minimized; finally, the immunosensor did not show cross-reactions with other common pesticides like organophosphates, pyrethroids, and neonicotinoids.

**Figure 4:**
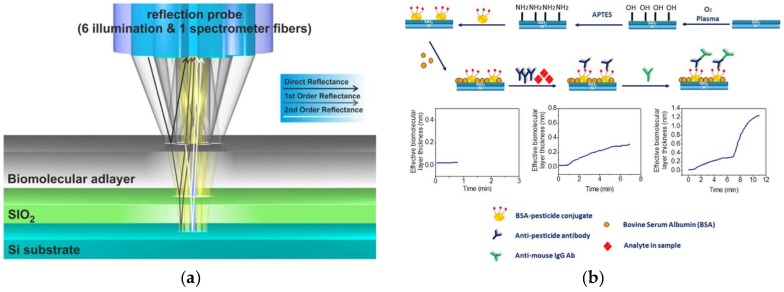
(**a**) White light reflectance spectroscopy principle of operation. (**b**) Schematic of the activation/biofunctionalization procedure of the chip surface and the competitive assay for the determination of pesticides (Reprinted with permission from [107]).

For the label-free and real-time detection of chlorpyrifos in water and wine samples, Koukouvinos et al. [107] developed a biosensor based on white light reflectance spectroscopy (WLRS) (Figure 4a) and a competitive immunoassay format (Figure 4b). The LOD was 0.6 ng/mL with an analysis time of about 10 min. Reflectometric interference spectroscopy systems show advantages with respect to other optical transduction principles, due to an user-friendly instrumentation, simplicity in the optical setting at low-cost. WLRS is based on the homogeneous illumination of the biosensor surface using optical fibers arranged circularly around the modified chip surface. The reflected light from the surface is then collected by an optical fiber positioned at the center of illumination circle. The interaction of light with the substrate produces the interference fringes. The latter can be shifted to higher wavelengths upon the increase of the biomolecular layer thickness during the assay due to the interaction between the biorecognition element and the target analyte.

Using a portable SPR biosensor Mauriz et al. [108] determined the presence of chlorpyrifos and carbaryl in natural surface water samples. The LODs were 0.05 and 0.9 ng/mL for chlorpyrifos and carbaryl, respectively. To avoid turbidity, the real samples from tap water, river water and groundwater were filtered; these samples and distilled water were spiked with the pesticide to evaluate potential effects on SPR immunoassays. The cross-reactivities evaluated with chlorpyrifos metabolites and related compounds was negligible for anti-chlorpyrifos-Ab and there are no interferences in the detection with different natural waters.

#### 3.2.2. Electrochemical Immunosensor

**Figure 5:**
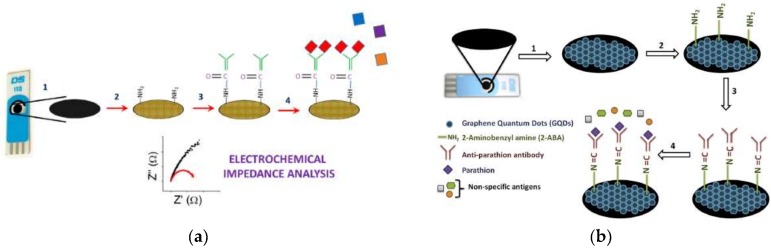
(**a**) Graphene modified screen-printed electrodes. (**b**) Graphene quantum dot modified SPE (reprinted with permission from [60,110]).

Martini et al. reported the detection of parathion in olive oil [58] and sunflower oil [109], respectively, through an amperometric immunosensor working in organic solvent mixtures (OPIEs). A Clark electrode was used as transducer and hapten-horseradish peroxidase as marker, with Ab sites immobilized onto a polytetrafluoroethylene (PTFE) membrane. The LOD was of 1.5 ng/mL (0.5 × 10^−8^ M) and a linear range of 3–700 ng/mL (1.0 × 10^−8^–2.5 × 10^−6^ M). Similarly, Mehta et al. developed a graphene modified screen-printed electrodes (SPE) [60] and a graphene quantum dot (GQD) modified SPE [110] immunosensors for the detection of parathion. In both cases the SPE was modified using amino functionalization to fix the anti-parathion Ab. Analysis was measured by electrochemical impedance spectroscopy (EIS) in a three-electrode cell (Figure 5). In the first case, a LOD of 52 pg/L and a linear range of 0.1–1000 ng/L was obtained. For GQD the LOD was lowered to 46 pg/L and the linear range was extended from 0.01–10 ng/L. In order to evaluate the specificity of the technique, the authors assayed a control in a blank sample with other organophosphate pesticides (paraoxon, malathion, and monochrotophos) in elevated ratio concentrations. The sensors show high specificity to parathion without response to the other pesticides.

**Figure 6:**
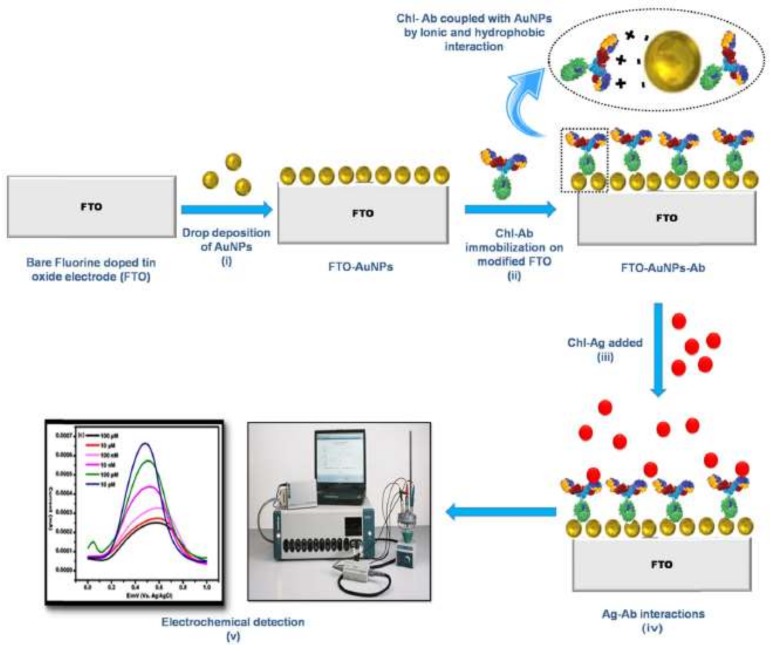
Anti-chlorpyrifos immobilized on fluorine-doped tin oxide with gold nanoparticles to direct determination of chlorpyrifos (reprinted with permission from [59]).

Talan et al. [59] developed an electrochemical immune-nanosensor for the detection in apple and cabbage of chlorpyrifos, using a fluorine-doped tin oxide (FTO) electrode modified with gold nanoparticles (AuNPs) and anti-chlorpyrifos antibodies (Figure 6). The linear range of the sensor was from 0.35 pg/L (1 fM) to 3.5 mg/L (1 μM) with LOD to apple and cabbage of 3.5 μg/L (10 nM).

Table 1 shows the analytical features of some electrochemical immunosensors for organophosphates and carbamates determination reported in literature. Although the reported immunosensors were very specific for the pesticides under study (no significance interference detected when several pesticides were assayed simultaneously), careful control of some variables should be taken into account to get the best sensor performance, such as antigen dilution ratio, antibody dilution ratio, pH value, and incubation time because.

### 3.3. Neonicotinoids

Neonicotinoids is a class of systemic insecticides, which is absorbed by the roots and distributed to leaves, tissues, fruits, and flowers, acting on the nicotinic acetylcholine receptors (nAChRs) [117]. Their use has grown a lot in recent decades, since their introduction to the market in the 1990s, becoming currently the most widely used type of insecticide around the world with around 30% of total sales [118,119].

Neonicotinoids can persist for a long time in soil and water, with a half-life of up to three years [120]. The most used neonicotinods agents are: clothianidin, imidacloprid, nitenpyram, dinotefuran, acetamiprid, thiamethoxam, and thiacloprid. These molecules are considered contaminants because they have a high toxicological potential in non-target organisms such as terrestrial and aquatic animals [121] and even in humans and mammals [120,122], but the biggest risk that has been studied and reported is in bees [117,118,123]. EFSA has pronounced itself in this regard, confirms the risk in the exposition of these pesticides to the bees [124].

The MRL in the EU ant FAO are below to 2 mg/kg in the most cases [19,125]. For example, in the case of imidacloprid the MRL to cauliflower, apple, cereal grains, maize, tomato, and lettuce are equal or below to 0.5 mg/kg.

Due to their large distribution and increased use [126], it is necessary to have methods that facilitate the detection of these contaminants. Liquid chromatography–tandem mass spectrometry is generally used to determine a large number of neonicotinoids and metabolites [127], but as mentioned above it requires laborious sample treatments and high costs. Once again, immunosensors facilitate the detection of these pesticides, reducing costs and time aside from being practical, sensitive, and selective.

#### 3.3.1. Optical Immunosensors

In EU, the outdoor use of imidacloprid, clothianidin and thiamethoxam [128], is prohibited. However, several biosensing formats have been carried out. For example, imidacloprid has been detected by fluorescence originated from inner filter effect (IFE) between upconversion nanoparticles (UCNPs) and gold nanoparticles (AuNPs). In that report, a competitive immunoreaction was performed between imidacloprid and antigen-AuNPs for the binding to the antibody-UCNPs. The LOD was 0.79 ng/mL and the linear range was 1.39–335 ng/mL. Water samples, chinese cabbage, and honey [129] samples have been tested. Cross-reactivity assays showed that the immunosensor was affected by interference of imidaclothiz, clothianidin, and acetamiprid. Lee et al. [130] developed an easy, low-cost, and label-free detection technique for qualitative and semiquantitative analysis of imidacloprid, based on an indirect competitive immunoassay. A SPR biochip combined with a simple portable imaging setup for label-free detection of pesticides was reported with a LOD of 1 ng/mL.

An optical immunosensor was developed by Hirakawa et al. [131] for detection of clothianidin and nitenpyram based on SPR. The SPR immunosensor showed specific reactivity in the range of 6.7–27 ng/mL for clothianidin, 15–93 ng/ml for boscalid, and 7.3–62 ng/mL for nitenpyram. Cross-reactivity assays showed that the sensor could also detect dinotefuran. Interestingly, the inmunosensor can determine simultaneously boscalid mixed with another pesticide (dinotefuran, clothianidin, or nitenpyra).

#### 3.3.2. Electrochemical Immunosensor

**Figure 7:**
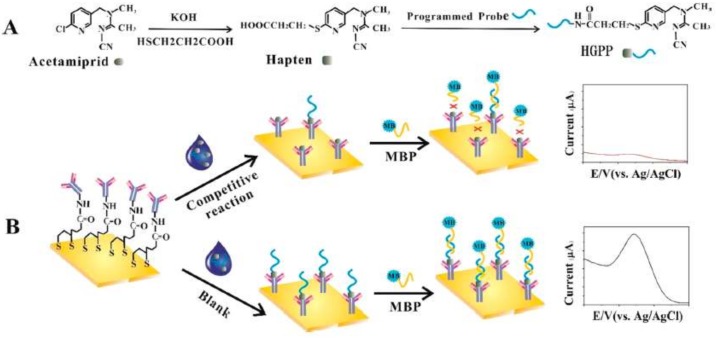
(**A**) Preparation of HGPP. (**B**) Competitive immunosensor assay for the detection of acetamiprid (reprinted with permission from [136]).

Some examples of electrochemical immunosensors are reported for the detection of neonicotinoids [132,133], especially with aptamers [134,135] but in our knowledge, only one work has published on immunosensor for detection of acetamiprid [136]. This work is based on hapten-grafted programmed probe (HGPP) that competes with acetamiprid for the sites of an antibody-modified gold electrode (Figure 7). The immunosensor showed highly sensitivity and selectivity in quantifying the amount of acetamiprid in spiked strawberry and cabbage extracts, with LOD of 3.2 ng/L and a linear range of 5–10^5^ ng/L. As depicted in Figure 7, control or blank experiments displayed a pronounced current response that in the presence of acetamiprid is significantly attenuated. Cross-reactivity experiments showed that six kinds of nontarget pesticides such as chlorpyrifos, methamidophos, imidacloprid, omethoate, 2,4-D, and carbofuran did not interfere with the signal, indicating selectivity against acetamiprid.

## 4. General Consideration and Conclusions

Pesticides have been classified as ubiquitous pollutants due to the intensive use, incorrect application, as well as the inadequate final disposal. Therefore, the detection of pesticides in environmental compartments and in food samples is of the highest relevance due to their toxicity. Immunosensors have some unique characteristics, important for their use as screening techniques in massive pesticide monitoring campaigns. To have them as a successful technology of routine application, immunosensors need to be improved to allow the detection of multianalytes in a fast and simple way, preferably in situ. The emerging trend for in situ analysis, whether at home or on remote location, originate the development of devices, which take advantage of integrated sampling and detection technologies. The originated characteristics mainly rely on portability, compactness and parallelization features. A lab-on-a-chip is a platform that integrates multiple laboratory functions on a single chip of only millimeters to a few square centimeters in size. Using these devices, the handling of extremely small fluid volumes is possible, even down to less than picoliters. The precise control of the movement of small volumes of liquid through the channels, can be achieved by pressure or electrokinetic forces. Some interesting examples can already be found in the literature [137,138]. Another important issue that can be easily covered by microfluidics is the possibility of multiplexed analysis.

An important point in the development of improved devices is the use of nanomaterials, with their ability to amplify the signal and of providing a more effective environment for the anchoring of the biological element. Additionally, the use of nanomaterials would potentially help in the miniaturization approach.

The literature reviewed in this paper demonstrates that immunosensors are capable of characterizing and quantifying pesticides in many kinds of environmental and food samples. Commercialization of immunosensors has not yet maintained pace with the large amount of research activity. This phenomenon has multiple reasons. The commercialization of new devices is retarded by both cost and technical issues. Nevertheless, it is important to continue working on these technologies to allow a successful transition from the laboratory research to the market.

## Figures and Tables

**Table 1:** Some examples of electrochemical immunosensor for chlorpyrifos and carbofuran determination.

Immunosensor	Compound Detected	Detection Limit	Linear Range	Matrix	Reference
Magnetic nanoparticles-modified	Chlorpyrifos	0.01 µg/mL	0.01–10 µg/mL	Agricultural products	[111]
Graphene nanocomposites and hollow gold nanospheres	Chlorpyrifos	0.052 ng/mL	2–150 µg/mL	Vegetable samples	[112]
Interdigitated array microelectrodes	Chlorpyrifos	0.014 ng/mL	0.001–10 µg/mL	Chives, lettuce and cabbage	[113]
Microfluidic chip	Chlorpyrifos	0.5 ng/mL	0.5–500 ng/mL	Chives, lettuce and pakchoi	[114]
Gold electrode	Carbofuran	0.1 ng/mL	0.1–1000 ng/mL	Agricultural and environmental samples	[115]
Glassy carbon electrode	Carbofuran	1 ng/mL	0.001–100 µg/mL	Vegetable samples	[116]
